# Multimodal Assessment of Schizophrenia and Depression Utilizing Video, Acoustic, Locomotor, Electroencephalographic, and Heart Rate Technology: Protocol for an Observational Study

**DOI:** 10.2196/36417

**Published:** 2022-07-13

**Authors:** Robert O Cotes, Mina Boazak, Emily Griner, Zifan Jiang, Bona Kim, Whitney Bremer, Salman Seyedi, Ali Bahrami Rad, Gari D Clifford

**Affiliations:** 1 Department of Psychiatry and Behavioral Sciences Emory University School of Medicine Atlanta, GA United States; 2 Animo Sano Psychiatry Durham, NC United States; 3 Department of Biomedical Informatics Emory University School of Medicine Atlanta, GA United States; 4 Department of Biomedical Engineering Georgia Institute of Technology Atlanta, GA United States; 5 Visual Medical Education Emory School of Medicine Atlanta, GA United States

**Keywords:** digital biomarker, machine learning, computer vision, schizophrenia, depression, multimodal, technology, acoustic, heart rate, biomarker

## Abstract

**Background:**

Current standards of psychiatric assessment and diagnostic evaluation rely primarily on the clinical subjective interpretation of a patient’s outward manifestations of their internal state. While psychometric tools can help to evaluate these behaviors more systematically, the tools still rely on the clinician’s interpretation of what are frequently nuanced speech and behavior patterns. With advances in computing power, increased availability of clinical data, and improving resolution of recording and sensor hardware (including acoustic, video, accelerometer, infrared, and other modalities), researchers have begun to demonstrate the feasibility of cutting-edge technologies in aiding the assessment of psychiatric disorders.

**Objective:**

We present a research protocol that utilizes facial expression, eye gaze, voice and speech, locomotor, heart rate, and electroencephalography monitoring to assess schizophrenia symptoms and to distinguish patients with schizophrenia from those with other psychiatric disorders and control subjects.

**Methods:**

We plan to recruit three outpatient groups: (1) 50 patients with schizophrenia, (2) 50 patients with unipolar major depressive disorder, and (3) 50 individuals with no psychiatric history. Using an internally developed semistructured interview, psychometrically validated clinical outcome measures, and a multimodal sensing system utilizing video, acoustic, actigraphic, heart rate, and electroencephalographic sensors, we aim to evaluate the system’s capacity in classifying subjects (schizophrenia, depression, or control), to evaluate the system’s sensitivity to within-group symptom severity, and to determine if such a system can further classify variations in disorder subtypes.

**Results:**

Data collection began in July 2020 and is expected to continue through December 2022.

**Conclusions:**

If successful, this study will help advance current progress in developing state-of-the-art technology to aid clinical psychiatric assessment and treatment. If our findings suggest that these technologies are capable of resolving diagnoses and symptoms to the level of current psychometric testing and clinician judgment, we would be among the first to develop a system that can eventually be used by clinicians to more objectively diagnose and assess schizophrenia and depression with the possibility of less risk of bias. Such a tool has the potential to improve accessibility to care; to aid clinicians in objectively evaluating diagnoses, severity of symptoms, and treatment efficacy through time; and to reduce treatment-related morbidity.

**International Registered Report Identifier (IRRID):**

DERR1-10.2196/36417

## Introduction

### Background

Mental disorders represent the second most common cause of years of life lived with disability worldwide [1]. Among mental illnesses, depression and schizophrenia represent the first and third highest contributors to years of life lived with disability, respectively, with over 200 million people suffering from either condition across the globe [2]. Schizophrenia is one of the most severe psychiatric disorders and affects self-image, physical health, employment, and social life [3]. While the cost of both conditions is tremendous, schizophrenia is particularly dramatic, with reports estimating an average of 14.5 to 28.5 years of life lost in those who suffer with the disorder [4,5]. The profound impact on the individual notwithstanding, these disorders are also of tremendous public concern. The World Health Organization estimates that the cost of schizophrenia reaches 2.6% of health care spending in high-income nations [6]. In the US, studies have found that the costs of depression and schizophrenia reach an annual US $210 billion and US $155 billion, respectively [7,8]. To put that in perspective, the Center for Medicare and Medicaid Services reported that in 2016, annual health care expenditure was US $3.3 trillion [9], meaning that depression and schizophrenia accounted for 11.1% of annual American health care costs. The largest contributors to these costs include unemployment, caregiving, and inpatient care [7,8]. Limiting the individual- and population-level impact of these disorders has been the subject of much research and has been a focus of the health care system.

Current evidence suggests that early identification and optimized treatment of depression and schizophrenia improves outcomes and reduces illness progression [10-14], which may consequently reduce societal costs. Unfortunately, the duration of untreated disease can be long. For example, in the Recovery After an Initial Schizophrenia Episode Early Treatment Program study, the median duration of untreated psychosis was 74 weeks [15]. Multiple factors contribute to delays in the diagnosis of these conditions, including limited access to psychiatric care [16,17]. Even in high-income nations, such as the US, access to specialty care is limited, with reports finding that up to 20.1% of adults seeking care do not receive treatment meeting their needs [18].

At present, depression and schizophrenia are diagnosed through the subjective clinical evaluation of signs and symptoms established by the Diagnostic and Statistical Manual of Mental Disorders (DSM-5) [19] or the International Classification of Diseases, 10th revision [20]. Structured interviews can be used to improve diagnostic accuracy, but are infrequently used in clinical practice [21]. While at their extremes these conditions are far from nuanced, symptoms can be subtle in the early phases [22]. In such instances, disorder identification requires extended interviews, gathering of collateral information, and a high degree of expertise from the interviewer. Unfortunately, the current and likely future state of the behavioral health system significantly restricts patient access to the necessary extended interviews by the appropriate experts. In fact, a 2017 report estimated that the supply of psychiatrists in the United States would decrease by 20% over the next decade, despite increasing demand over the same period [23]. As a consequence, behavioral health care is increasingly being managed by primary care providers who have neither the time nor the expertise to evaluate subtle variations in certain behavioral health conditions [24]. This represents an opportunity for the integration of technological support tools to improve patient access to quality care.

### Current Digital Biomarker Research

The development of easy-to-use, objective clinical tools to aid clinicians in the diagnosis and evaluation of mental illness has the potential to limit the impact of these illnesses on patients and on society. The cost of powerful computing hardware has fallen, and improvements in the field of computer science and health care suggest that various types of computer sensors and recording hardware could be used to aid in the assessment and diagnostic prediction of mental illness [25-30]. Research groups have demonstrated the efficacy in numerous mental health populations of these technologies, which include computer vision for distinguishing phases of depression [25,26], schizophrenia [27], and cognitive impairment [31], actigraphy for the differentiation of patients with schizophrenia from controls [28,29], and heart rate monitoring for distinguishing patients with schizophrenia or posttraumatic stress disorder from controls [28-30]. This research has demonstrated that with heart rate variability and actigraphic assessments alone, patients with schizophrenia may be differentiated from controls with up to 95.3% accuracy [28,29]. Other groups have similarly demonstrated the efficacy of each of these technologies in schizophrenia.

Few studies, however, have been conducted to assess variations in patients with depression or schizophrenia and control subjects with video technology, although some studies have observed statistically significant differences between schizophrenia and control groups in certain combinations of facial action clustering [32-34]. Furthermore, the extent of facial action expressivity has been demonstrated to be well correlated with clinical assessments of symptoms of schizophrenia [32,34,35]. Similar to facial expressions, video recording assessments have also allowed for differentiation of eye gaze behavior between subtypes of schizophrenia [36]. Similarly, actigraphic data has been demonstrated to be of value, with one group demonstrating a correlation between actigraphy recordings and changes in patient clinical conditions and drug regimens [37,38]. In a separate study of 25 patients with psychosis, the same research group assessed variations in motor richness, typicality, and consistency in patient subtypes with “high-positive,” “high-negative,” and “low-level” symptoms, finding differences in richness and typicality, but not in consistency, between the subgroups [39]. Other groups have similarly found computerized voice and speech assessment to be clinically useful, with findings suggesting that voice pause and, to some extent, pitch can be discriminative of schizophrenia [40]. Furthermore, it has been demonstrated that speech coherence assessments have discriminative capacity in differentiating patients with schizophrenia from controls [41] and in predicting progression from prodrome to psychosis [42].

Past research groups have also found that electroencephalographic (EEG) recordings can accurately classify schizophrenia. For instance, one group found that EEG recordings could classify schizophrenia with 91.5% to 93.9% accuracy [43]. Multiple research groups have also studied automated EEG-based diagnosis of depression. In a review of several computer-aided diagnostic methods based on EEG data [44], one group showed that nonlinear dynamical analysis of EEG data is a promising approach for the differentiation of normal and depressed subjects [45]. Moreover, depression detection has been demonstrated using 3-electrode EEG-based analysis using wavelet transformation, feature selection, and multiple classification algorithms [46]. More recently, there has been a considerable number of deep neural network approaches [47,48]. However, generally, these approaches use small study populations and overfit without proper sampling and stratification.

Combining biomarkers may be a promising approach to improving diagnostic precision and treatment options [49,50] and may be applicable to the study of digital biomarkers. Approaches using multiple digital inputs have been used to differentiate individuals with depression from those without depression [51] and also to predict mood states (eg, depression, mania, and hypomania) in patients with mood disorders [52]. While research groups have assessed the role of these technologies in differentiating limited subtypes of schizophrenia, in addition to differentiating patients with schizophrenia from control groups, few have assessed the combination of these technologies in the assessment of schizophrenia, including the potential of combined technologies to improve predictive efficacy in differentiating patients with schizophrenia from controls, discriminate schizophrenia from other mental illnesses, predict illness severity, assess symptom change over time, and assess illness-related movement disorders with video technology.

### Research Aims and Hypotheses

We present a research protocol to assess the efficacy of a multimodal sensor system combining video, audio, actigraphy, noninvasive EEG, and heart rate monitoring to assess differences in individuals with schizophrenia and unipolar major depressive disorder and controls (patients with no history of mental illness in the preceding year). The data collected from the subjects will be utilized to develop a machine learning model to evaluate the presence, severity, and possible subtypes of schizophrenia and depression. We seek to assess the performance of the developed model and its ability to differentiate schizophrenia from depression and control groups based on high-value input features from different modalities. We hypothesize that the predictive model will discriminate between schizophrenia, depression, and control groups. For the depression group only, we will evaluate within-group preprocessed outputs of the sensor data to discriminate depression severity scores using the Patient Health Questionnaire-9 (PHQ-9) [53] and the Clinical Global Impression (CGI) [54], commonly utilized rating scales to measure illness severity. For the schizophrenia group, we will evaluate within-group preprocessed outputs of the sensor data to discriminate severity scores using the Positive and Negative Syndrome Scale (PANSS) [55], Clinician-Rated Dimensions of Psychosis Symptom Severity in Patients with Schizophrenia (CRDPSS) [19,56] and the CGI. Finally, we will evaluate sensor sensitivity to within-subject outcome changes over time for both groups.

## Methods

### Participants

The research assessments will be conducted at the Grady Outpatient Behavioral Health Clinic, which is part of the broader Grady Health System, a metropolitan safety-net hospital in Atlanta, Georgia. The study seeks to recruit 50 individuals with schizophrenia, 50 individuals with unipolar major depressive disorder, and 50 controls without a prior history of mental illness.

### Inclusion and Exclusion Criteria

The 3 groups of participants (aged 18 years or older) will include outpatients with a DSM-5 diagnosis of schizophrenia or a DSM-5 diagnosis of major depressive disorder and individuals with no mental health diagnosis (as controls). All diagnoses will be confirmed by the Mini International Neuropsychiatric Interview (MINI) [57]. All subjects must have the ability to consent to participation. Individuals that have a legal guardian may participate in the study with the guardian’s signed consent. Participants recruited digitally must have access to a webcam (either on a desktop, tablet, or cell phone) and have internet connectivity with at least 2 to 4 megabytes per second upload speed. Subjects who do not have the capacity to consent to participation, who meet the criteria for nonschizophrenia psychotic illness (eg, schizoaffective disorder, mood disorder with psychotic features, or substance-induced psychotic disorder), whose differential diagnosis includes an active substance-induced mental illness, who present as unreliable with the equipment, who cannot participate in full assessments, or whose differential diagnosis includes a personality disorder will be excluded from the study. Those who are not native English speakers will be excluded from the study. The study team will make it clear that mental health treatment is not provided in this research study. If individuals (who are either included or excluded from the study) express an interest in connecting to local mental health resources, the team will make reasonable attempts to help them do so. If subjects present with signs or symptoms suggestive of a mental health emergency, a clinician (study author ROC) will be contacted for subject evaluation to determine whether emergency care is necessary.

### Procedure

All participant groups will be recruited (1) from a database of interested research participants from prior research studies, from clinician referrals, and from respondents to a general research interest form provided in the outpatient waiting rooms of the Grady Outpatient Behavioral Health Clinic; (2) through a regional digital recruitment strategy, part of ResearchMatch (of which Emory University is an institutional participant), that will target the metro Atlanta area for in-person interviews and the entire United States for remote or telehealth interviews; and (3) through a digital recruitment strategy based on Amazon Mechanical Turk (Amazon Inc) in which individuals that respond to a short questionnaire will be able to reach out to the study team via email if they are interested in participating in the study.

**Figure 1:**
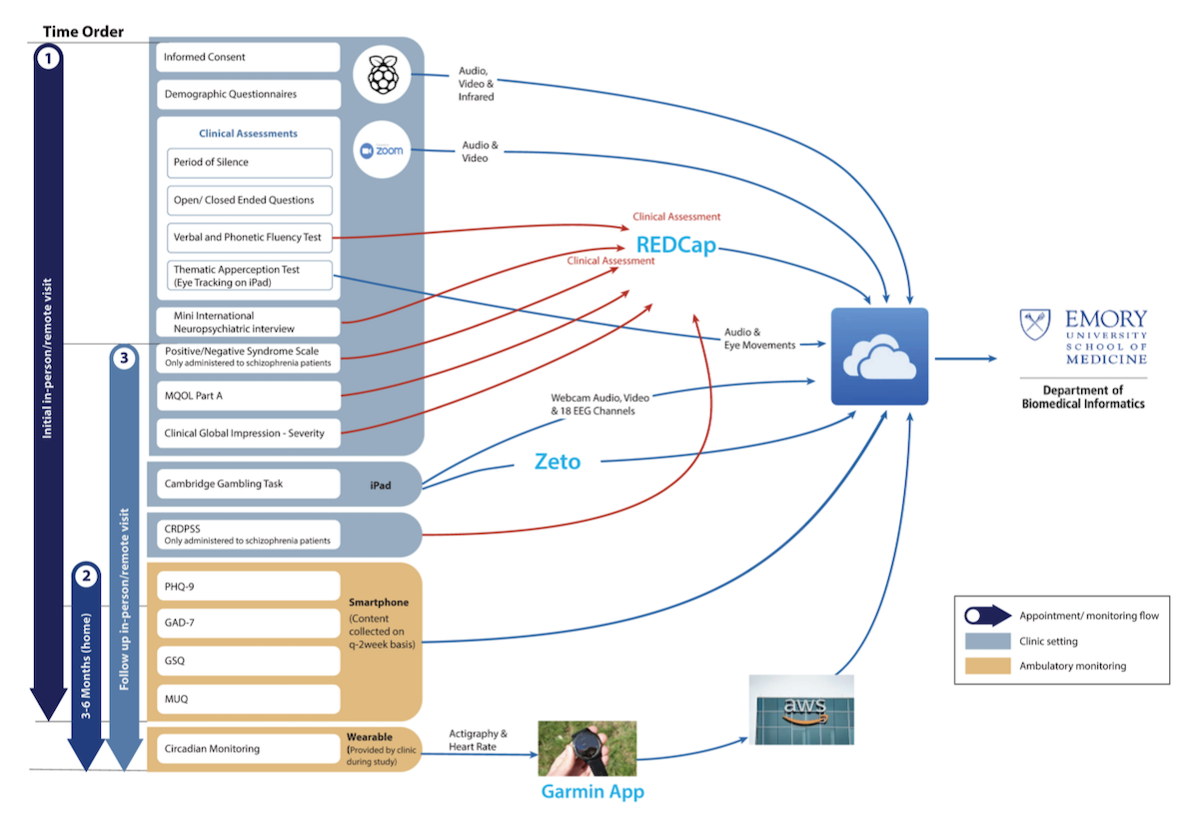
Initial interview data collection process. MQOL Part A: McGill Quality of Life Questionnaire-Revised Part A; CRDPSS: Clinician-Rated Dimensions of Psychosis Symptom Severity in Patients with Schizophrenia; PHQ-9: Patient Health Questionnaire-9; GAD-7: General Anxiety Disorder-7; GSQ: General Symptom Questionnaire; MUQ: Medication Utilization Questionnaire; EEG: electroencephalogram.

The schizophrenia and control groups that participate in person will be interviewed at 2 time points (the initial encounter and a second encounter, 3 to 6 months after the initial encounter) for all measures, as indicated below. Figure 1 shows a schematic of the initial interview data collection process. The depression, schizophrenia, and control groups that participate remotely will only be interviewed at baseline and will not have a follow-up assessment for any of the measures indicated below. Given the established need for continuous assessment of heart rate variability and locomotor activity to adequately discriminate patients with schizophrenia from controls [28], all in-person (schizophrenia and control) outpatient populations will be assessed for heart rate variability and locomotor activity for the 3 months leading up to second appointment. This study involves the collection of video, voice and speech, actigraphic, pulse oximetry, heart rate, EEG, questionnaire, interview, and available clinical data only.

All assessments will take place over Zoom (Zoom Inc), a secure, encrypted, telehealth platform that is compliant with the Health Insurance Portability and Accountability Act of 1996 (HIPAA). In-person assessments will consist of an interviewer conducting an interview via Zoom on a computer, with the participant on a different computer located in an adjacent room in the research suite. For remote assessments, the interviewer will be physically located at the research suite and the participant will be on their own computer at the location of their choice (usually their home). Due to the COVID-19 pandemic, assessments will be conducted in person when local case counts are low and the research team is safely able to complete interviews with social distancing measures in place and appropriate personal protective equipment (PPE).

In-person participants will be interviewed twice, 3 to 6 months apart, and offered a US $30 honorarium at the completion of study visit 1 and a US $30 honorarium at the completion of study visit 2. Between visits the participants may complete a battery of assessments offered every 2 weeks on their devices, for which they will be compensated US $5 for each battery. Remote participants will be interviewed once and offered a US $30 honorarium at the completion of the study visit. Individuals who participate in the study but are unable to complete it will be offered a US $10 honorarium. Those subjects screened and determined to not meet the eligibility criteria will not be offered compensation.

### Ethics Approval

The study was approved by the Emory University Institutional Review Board in November 2018 (IRB00105142) and the Grady Research Oversight Committee in January 2019 (00-105142).

### Measures

The schedule of assessments is found in Table 1 for the in-person assessments and Table 2 for the remote assessments. For subjects with schizophrenia in ambulatory treatment, monitoring will occur in one of two ways: the subjects will be evaluated at the initial encounter and 3 months after the initial encounter in person, or they will be evaluated remotely once via Zoom.

**Table 1:** Schedule of assessments for in-person visits.

Assessments	Visit 1	Biweekly^a^	Visit 2 (3-6 months)^a^
Informed consent	✓		
Semistructured interview	✓		✓
Demographic assessment	✓		
Sociodemographic assessment	✓		
Mini International Neuropsychiatric Interview	✓		
Positive and Negative Syndrome Scale^b^	✓		✓
McGill Quality of Life Questionnaire-Revised Part A	✓		✓
Clinician-Rated Dimensions of Psychosis Symptom Severity in Patients with Schizophrenia^b^	✓		✓
Clinical Global Impression-Severity	✓		✓
Clinical Global Impression-Improvement			✓
Cambridge Gambling Task^a,c^	✓		✓
Patient Health Questionnaire-9^d^	✓	✓	✓
Generalized Anxiety Disorder-7^d^	✓	✓	✓
General Symptom Questionnaire^a,d^	✓	✓	✓
Medication Utilization Questionnaire^a,d^	✓	✓	✓
Facial expressivity and eye gaze	✓		✓
Voice and speech data collection	✓		✓
Electroencephalography^a,c^	✓		✓
Actigraphy and heart rate (continuous)^a,c^	✓	✓	✓

^a^Depression group excluded.

^b^Only administered for schizophrenia group.

^c^Only for in-person visits (ie, excluding subjects who were recruited digitally).

^d^Completed on participant devices for the schizophrenia and control groups only.

**Table 2:** Schedule of assessments for remote visits.

Assessments	Visit 1
Informed consent	✓
Semistructured interview	✓
Demographic assessment	✓
Sociodemographic assessment	✓
Mini International Neuropsychiatric Interview	✓
Positive and Negative Syndrome Scale^a^	✓
McGill Quality of Life Questionnaire-Revised Part A	✓
Clinician-Rated Dimensions of Psychosis Symptom Severity in Patients with Schizophrenia^a^	✓
Clinical Global Impression-Severity	✓
Patient Health Questionnaire-9	✓
General Anxiety Disorder-7	✓
Facial expressivity and eye gaze	✓
Voice and speech data collection	✓

^a^Only administered for schizophrenia group.

The initial assessment will include demographic and clinical information that will be collected via self-reporting. Clinical information will include information about self-reported psychiatric and medical comorbidities and current medications. The evaluations will include a clinical record review; a battery of psychometric tests, including a semistructured group developed interview that will include the Thematic Apperception Test (TAT) [58], the Semantic Fluency Task [59], and the Phonetic Fluency Task [60] (more details are available in Multimedia Appendix 1); and demographic and sociodemographic questionnaires, including the MINI, the McGill Quality of Life Questionnaire-Revised Part A (MQOL Part A) [61], the Clinical Global Impression Severity (CGI-S) and Clinical Global Impression Improvement (CGI-I) scales (in-person only) [54], PHQ-9 [53], the General Anxiety Disorder-7 (GAD-7) [62], and the Cambridge Gambling Task (CGT) (in-person only) [63,64]. The evaluations will also include audiovisual recordings, and the in-person evaluations will include pulse oximetry recordings taken during the entirety of the interview and electroencephalographic recordings taken during selected points of the interview, including the CGT. Actigraphy and heart rate recordings will be assessed for the 3-month period between the patient interviews for applicable participants. Only subjects with schizophrenia will receive the PANSS and CRDPSS. Further description of the rating scales used in the study can be found in Multimedia Appendix 1.

Depression subjects will only be evaluated once. The evaluations will include a clinical record review; a battery of psychometric tests, including a semistructured group developed interview; and demographic and sociodemographic questionnaires, including the MINI, MQOL Part A, CGI-S, PHQ-9, GAD-7, and CGT (in-person only). The evaluations will also include audiovisual recordings, and in-person evaluations will include pulse oximetry and electroencephalographic recordings.

For control subjects, monitoring will occur in one of two ways: the subjects will be evaluated at the initial encounter and 3 months after initial encounter in person, or they will be evaluated remotely once via Zoom. Evaluation will include clinical record review; a battery of psychometric tests, including a semistructured group developed interview; and demographic and sociodemographic questionnaires, including the MINI, MQOL Part A, CGI-S, PHQ-9, GAD-7, and CGT (in-person only). Audiovisual, pulse oximetry (in-person only), and electroencephalographic recording (in-person only) will be performed for the entirety of the interview and actigraphy and heart rate recording will be assessed for the 3-month period between interviews for applicable patients. All evaluations will be conducted in person during times when visits can be completed safely with social distancing measures and appropriate PPE use; otherwise, they will be conducted remotely via Zoom.

### Statistical Analysis, Model Development, and Data Integration

The data will undergo a descriptive statistical analysis and assessment of classifier and regressor capacity to predict diagnostic and psychometric scores, respectively. Descriptive statistical assessment of the collected data will be conducted. Diagnostic and psychometric score prediction will be conducted utilizing extracted features from the collected data. Sample data descriptions including demographics, clinical diagnosis, and psychometric scores will be analyzed. Cross-group variations in psychometric scores will be reported and analyzed via inferential statistical methods (2-way *t* testing).

Recordings of the interviews will be captured using Zoom at various resolutions, depending on the interviewee’s camera and network conditions. The participants will be asked to sit close enough to the camera that the interviewer can clearly see their face, with a minimum of 10% of the participant’s face filling the viewable screen. Three different simultaneous audio files will be generated, corresponding to the bidirectional conversation, the interviewee only, and the interviewer only. This separation will enable the research team to isolate the individual speaker, and also to evaluate how the system might work in a single-microphone environment.

Video analysis of the full face involves both the dynamics of facial action units [65,66], and full-face emotion interpretation using a deep convolutional neural network. We will follow our earlier work [67] and use an extensive pretrained network to identify the amount of time spent in 1 of 7 basic emotions (including neutrality, happiness, surprise, anger, sadness, fear, and disgust), and then use transfer learning to retrain the network on the new subjects using severity of depression or schizophrenia as a new target.

The audio will be analyzed in several ways. First, we will compare the outputs of two HIPAA-compliant commercial services for transcription of the audio recordings: the Otter.ai (Otter.ai Inc) service through Zoom’s transcription service and Amazon Transcribe (Amazon Inc). At the time of writing, no information on the performance of Otter.ai is available publicly. Amazon Transcribe has been shown to have word error rates of 10% to 20%, with small biases for gender and medical condition [68]. We will match the separate and combined transcripts for each service and compare a subsample to expert overreads to identify error rates. Natural language tools, such as the Linguistic Inquiry and Word Count dictionary [69], Word2Vec [70], and other related natural language processing tools [71-73] will be used to associate word- and sentence-level features with severity of depression or schizophrenia. These tools will be utilized throughout the interview and specifically for certain elements of the semistructured group developed interviews, including the TAT, the Semantic Fluency Task, and the Phonetic Fluency Task. Natural language processing measures analyzed from the TAT have been used to discriminate between patients with a first episode of psychosis and controls [74]; generally, semantic verbal fluency is more affected than phonemic fluency in schizophrenia [75,76]. We will also test the performance as a function of the transcription error rate. The nonsemantic content of the speech will be determined from the pitch, relative temporal ordering, duration information, and the dynamics of the audio recordings [25]. Here, statistical analysis, standard machine learning (eg, random forests), and deep neural networks will be utilized.

EEG recordings will be made using noninvasive scalp leads attached during the CGT [64,77] and baseline measurements. After the EEG device is in place and immediately prior to the CGT, the participant will undergo a 1-minute evaluation in a restful, eyes-open state. Following this, the participant will undergo an additional 1-minute evaluation in a restful, eyes-closed state. EEG recordings will be conducted only during in-person visits. The EEG analysis will involve standard approaches, such as EEG band–related signal (delta, theta, alpha, and beta) analysis [78] and state-of-the-art deep neural network analysis [79,80]. Different statistical descriptors, Hjorth parameters [81], and synchrony [82] will be analyzed for the EEG band–related signals. The recurrent neural networks [83,84] will be utilized for capturing the dynamical properties of the EEG signal. The heart rate and the actigraphy data recorded from the wearable devices will be analyzed with the multiscale network analysis developed in Reinertsen et al [85].

As described above and shown in Figure 1, data from all modalities will be captured, stored, and processed in HIPAA-compliant environments. More specifically, all the data will be stored in Emory University’s OneDrive server (Microsoft Corp) and will be processed in the high-performance computing cluster located in the Department of Biomedical Informatics at Emory University. Access to the identifiable personal data will be limited to the personnel approved by the institutional review board at Emory University.

In addition to the feature extraction and analyses within each modality, we aim to investigate the interaction between features from different modalities and fuse them together to build a multimodal machine learning model to estimate the presence and the severity of depression and schizophrenia. For interaction, we will check the Pearson correlations between features and between the predictions made with features from each single modality. Additionally, we will use dynamic time warping and general dynamic time warping [86] to check the similarity and alignment between time series of features from different modalities. For integration, we will use both model-agnostic and model-based approaches. For instance, we will test late fusion of the predicted results using features from each modality and early direct fusion of the features extracted from all modalities. We will also use temporal models, such as multi-view long short-term memory networks [87] and multimodal transformers [88].

## Results

**Figure 2:**
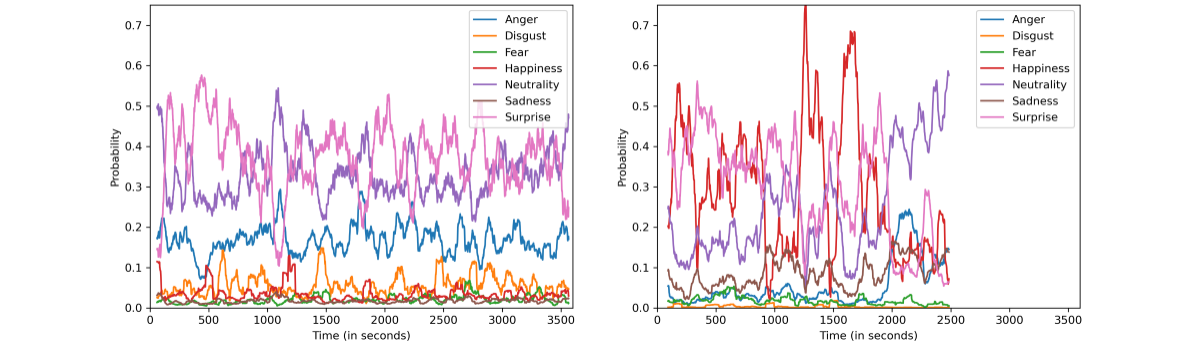
Measures of facial expression when comparing a patient with schizophrenia (left) to a control subject (right).

Data collection began in July 2020 and is expected to continue through December 2022. We present some preliminary data here, highlighting a comparison of the fluctuation of emotions using computer vision. Figure 2 visualizes the level of neutrality, happiness, surprise, anger, sadness, fear, and disgust of a patient with schizophrenia (on the left) versus a control (on the right) during the interview. Distinct differences could be found between these 2 participants, including the average strength and average length of different emotions and how fast they switched between emotions. In the case of these 2 participants, the patient with schizophrenia was found to have a longer duration of neutrality, shorter duration of happiness, and a faster change between emotions. Statistical tests need to be performed on the final, larger groups to provide concrete evidence on whether there indeed exist group-level differences.

## Discussion

This study aims to advance current progress in the use of state-of-the-art technology for assisting clinical psychiatric assessments by using a novel multimodal sensing system.

### Strengths

The in-person recruitment site, Grady Health System, allows access to a racially and ethnically diverse group of potential participants. Furthermore, the study methods will allow for data collection to continue irrespective of local case fluctuations in COVID-19 infection rates. The study team will be able to recruit and evaluate participants in person or pivot to virtual recruitment and data collection using a national recruitment database.

### Limitations

This study has a number of limitations. Larger data sets will be needed and the model will need to be prospectively validated. Additionally, remote participation is only available to those who have access to the internet, a video camera, and a microphone. The quality of these recordings is subject to variation based upon the devices accessible to the participants and the areas where they are located, which may impact the quality of the recordings and limit recruitment efforts. The protocol has provided an option for subjects to participate in person, which will allow the study team to better standardize the technology used. However, there may be differences between subjects who participate in their homes and those who travel to the research site to complete the interviews. The research site will provide a private room to complete the Zoom interview and will mimic, as much as possible, the environment of those participating remotely from their homes. Furthermore, the interviewers may be at risk of bias in their ratings, and their physical presence or absence may affect patient responses. All assessments will be recorded, which will allow for the verification of all ratings by the interviewers and the study psychiatrist. The interviewers will also follow a script to maintain as much between-subject consistency in the interviews as possible.

### Conclusions

If our findings suggest that these technologies are capable of resolving diagnoses and revealing symptoms at the same level as current psychometric testing and clinician judgment, we will be among the first in the world to have developed a clinical decision support system that can be used by expert and nonexpert clinicians for objectively diagnosing and tracking schizophrenia and depression over time. Such a tool would improve accessibility to care; aid clinicians in objectively evaluating diagnoses, severity of symptoms, and treatment efficacy; reduce treatment-related morbidity; and potentially empower patients to gain a deeper insight into their day-to-day symptoms and stressors to guide self-management.

## Appendix

Multimedia Appendix 1 Supplemental Materials.

## Abbreviations

CGI-I: Clinical Global Impression-Improvement
CGI-S: Clinical Global Impression-Severity
CGT: Cambridge Gambling Task
CRDPSS: Clinician-Rated Dimensions of Psychosis Symptom Severity in Patients with Schizophrenia
DSM-5: Diagnostic and Statistical Manual of Mental Disorders
EEG: electroencephalogram
GAD-7: General Anxiety Disorder-7
GSQ: General Symptom Questionnaire
HIPAA: Health Insurance Portability and Accountability Act of 1996
MINI: Mini International Neuropsychiatric Interview
MQOL Part A: McGill Quality of Life Questionnaire-Revised Part A
MUQ: Medication Utilization Questionnaire
PANSS: Positive and Negative Syndrome Scale
PHQ-9: Patient Health Questionnaire-9
PPE: Personal Protective Equipment
TAT: Thematic Apperception Test

## Data Availability

The data sets generated during or analyzed during the current study will not be publicly available because they contain protected health information, but deidentified subsets of the data will be made available from the corresponding author on reasonable request, if the resources to perform and validate the deidentification process are available.
