# Supplementary material for: Multimodal Assessment of Schizophrenia and Depression Utilizing Video, Acoustic, Locomotor, Electroencephalographic, and Heart Rate Technology: Protocol for an Observational Study
Source: JMIR Res Protoc. 2022 Jul 13;11(7):e36417. doi: 10.2196/36417 (PMC9330209; doi:10.2196/36417)
Supplement: Multimedia Appendix 1 [file resprot_v11i6e36417_app1.doc]

Supplemental Materials

Interview Questions

Please ask the subject the below questions (only note the words listed in the verbal fluency tasks):

“I am about to ask you some questions, some of these questions may seem out of place, please try to answer to the best of your ability. After I ask each question, I will not say anything and will give you at least 60 seconds to respond to the question. If you are done responding to the question expect me to wait 5-10 seconds before I say anything.” For questions 2-6, if the participant finishes and is silent for at least 10 seconds, move onto the next question. Do not transcribe responses from participant for questions 1-6.

1. “What is the current year?”

2. “How would you like to begin?”

3. “Tell me about yourself”

4. “Tell me about things that excite you”

5. “Tell me about your fears”

6. Thematic Apperception Test (TAT): “I am going to show you a picture and your task will be to make up a story. In your story, be sure to tell what has led up to the event shown in the picture, describe what is happening at the moment, what the characters are feeling and thinking, and then give the outcome. Tell a complete story with a beginning, middle, and end. Do you understand? Here is the card.” [Show TAT Card to Subject]

7. Semantic Fluency Task

8. Phonetic Fluency Task

Supplemental Materials

Further Description of Rating Scales

Mini International Neuropsychiatric Interview for the DSM-5 (MINI v 7.0.2 or equivalent)- Validated 17 module battery assesses for psychiatric disorder signs and symptoms meeting DSM-5 criteria. Will be used to assess subject psychiatric symptomatology and co-occurring mental illnesses at initial and final subject evaluations only.

Positive and Negative Syndrome Scale (PANSS)- Validated scale assessing 7 positive symptoms of schizophrenia, 7 negative symptoms of schizophrenia, and 16 general psychopathology items. Will be used to assess subject psychotic symptoms at initial and final evaluations only.

Clinician Rated Dimensions of Psychosis Symptom Severity (CRDPSS)- Validated 8 item tool for assessing core features of schizophrenia. Will be used to assess subject schizophrenia symptoms both at initial evaluation and follow up evaluation.

Clinical Global Impression Scale (CGI-Severity and CGI-Improvement scales)- Two validated 7-point scales assessing clinician impression of patient illness severity and impression of improvement of illness. Will be collected from clinicians at time of interviews.

McGill Quality of Life Questionnaire Part A- Single question assessing subject quality of life.

Patient Health Questionnaire-9 (PHQ-9)- A 9 item scale evaluating depression severity completed during the evaluation for subjects evaluated remotely and on the Moyo Health Network smartphone application, developed in-house, for subjects evaluated in-person.

General Anxiety Disorder-7 (GAD-7)- A 7 item scale evaluating anxiety severity completed during the evaluation for subjects evaluated remotely and on the Moyo Health Network smartphone application, developed in-house, for subjects evaluated in-person.

Baseline EEG Recordings – Prior to recording EEG during the Cambridge Gambling Task (CGT), participants will be asked to rest with their eyes open, while wearing the EEG device, for 1 minute. Following this, they will be asked to rest with their eyes closed, while still wearing the EEG device, for 1 minute.

Cambridge Gambling Task (CGT)- Evaluates decision making and risk-taking behavior. Participants will wear the EEG device while undergoing this assessment.

General Symptom Questionnaire (GSQ) – This is an in house, non-validated, self-reported assessment that assesses depression, mood, and psychotic symptoms completed on the Moyo Health Network smartphone application, developed in-house

Medication Utilization Questionnaire (MUQ)– This is a bi-weekly assessment of how often subjects have forgotten to take their medications as prescribed over the previous two weeks, completed on the Moyo Health Network smartphone application, developed in-house
